# Vaccines to Prevent Meningitis: Historical Perspectives and Future Directions

**DOI:** 10.3390/microorganisms9040771

**Published:** 2021-04-07

**Authors:** Mark R. Alderson, Jo Anne Welsch, Katie Regan, Lauren Newhouse, Niranjan Bhat, Anthony A. Marfin

**Affiliations:** Center for Vaccine Innovation and Access, PATH, Seattle, WA 98121, USA; jwelsch@path.org (J.A.W.); kregan@path.org (K.R.); lnewhouse@path.org (L.N.); nbhat@path.org (N.B.); aamarfin@path.org (A.A.M.)

**Keywords:** meningitis, meningococcus, pneumococcus, *Haemophilus influenzae*, Hib, group B streptococcus, conjugate vaccine

## Abstract

Despite advances in the development and introduction of vaccines against the major bacterial causes of meningitis, the disease and its long-term after-effects remain a problem globally. The Global Roadmap to Defeat Meningitis by 2030 aims to accelerate progress through visionary and strategic goals that place a major emphasis on preventing meningitis via vaccination. Global vaccination against *Haemophilus influenzae* type B (Hib) is the most advanced, such that successful and low-cost combination vaccines incorporating Hib are broadly available. More affordable pneumococcal conjugate vaccines are becoming increasingly available, although countries ineligible for donor support still face access challenges and global serotype coverage is incomplete with existing licensed vaccines. Meningococcal disease control in Africa has progressed with the successful deployment of a low-cost serogroup A conjugate vaccine, but other serogroups still cause outbreaks in regions of the world where broadly protective and affordable vaccines have not been introduced into routine immunization programs. Progress has lagged for prevention of neonatal meningitis and although maternal vaccination against the leading cause, group B streptococcus (GBS), has progressed into clinical trials, no GBS vaccine has thus far reached Phase 3 evaluation. This article examines current and future efforts to control meningitis through vaccination.

## 1. Introduction

Despite advances against individual pathogens, bacterial meningitis and sepsis remain public health challenges globally. Meningitis, characterized by inflammation of the meninges, is swift and severe and is associated with significant morbidity and mortality. Low- and middle-income countries (LMICs) suffer the greatest burden, with the African Meningitis Belt, a string of 26 countries from Senegal and The Gambia in the west to Ethiopia in the east, experiencing a disproportionate share of disease [1]. Bacterial meningitis epidemics are common in this region and many have been large-scale, threatening economic stability alongside human life. However, outbreaks and epidemics can occur globally [2,3]. There are an estimated 5 million cases of meningitis each year, with up to 300,000 deaths—nearly half of which are in children younger than five years of age (u5) [4]. Survivors are not always spared; a high proportion suffer long-term after affects including hearing loss, visual, physical, and cognitive impairment, and limb loss. Despite this sobering reality, progress against meningitis lags that of other vaccine preventable diseases [4].

The Global Roadmap to Defeat Meningitis by 2030, an initiative to raise awareness of bacterial meningitis as a public health problem and create a framework for addressing it, aims to reverse this trend. Critical goals include eliminating bacterial meningitis epidemics and reducing cases and deaths from the most significant causes of bacterial meningitis: *Haemophilus influenzae* type B (Hib), *Neisseria meningitidis* (meningococcus), *Streptococcus pneumoniae* (pneumococcus), and *Streptococcus agalactiae* (group B streptococcus (GBS)) [5]. Vaccines will play an essential role in preventing these diseases and fulfilling the roadmap vision. Effective vaccines exist and have been in use for years against some of these pathogens, and while there have been significant successes, there also remain significant challenges. As recognized in the World Health Organization (WHO) Immunization Agenda 2030, too many children have insufficient access to vaccines, driven in part by high prices for some of the most effective conjugate vaccines—resulting in limited availability in LMICs [6]. Moreover, existing vaccine formulations do not necessarily reflect the disease serogroups and serotypes most prevalent in the highest burden countries. And even in countries where vaccines are accessible, there is no standard approach to vaccination.

To defeat meningitis, it is critical that we advance new and better vaccines that will be affordable and accessible globally. This will not be easy, but past vaccine development efforts offer direction for future ones. From development of the first conjugate vaccine for humans to the groundbreaking Meningitis Vaccine Project (MVP) that effectively eliminated serogroup A meningococcal meningitis in Africa, the vaccine development landscape is rife with important lessons for developing and delivering vaccines to prevent meningitis [7].

Progress against the four key pathogens identified in the roadmap spans the vaccine development and delivery lifecycle. Hib conjugate vaccines (HibCVs), pneumococcal conjugate vaccines (PCVs), and meningococcal conjugate vaccines (NmCVs) have been in use for decades; vaccines against GBS are on the horizon. This article will explore the challenges, successes, and lessons learned through the development and introduction of meningitis vaccines—lessons critical for successful implementation of the roadmap and the strategy to defeat meningitis by 2030.

## 2. History and Status of Meningitis Vaccines

Hib, pneumococcus, meningococcus, and GBS are encapsulated bacteria that cause sepsis, meningitis, and other invasive and mucosal diseases [8]. Capsular polysaccharides are important virulence factors and have become the major vaccine target for all four pathogens. HibCVs, PCVs, and NmCVs are highly successful at preventing meningitis and other disease manifestations caused by these organisms. Conjugate vaccines against these bacteria not only protect against disease in multiple age groups, but also confer herd protection via reductions in pharyngeal carriage [9]. GBS is amenable to conjugate vaccine development but development thus far has targeted maternal immunization, given that the greatest disease burden occurs in the first three months of life [10].

HibCV was the prototype for targeting capsular polysaccharides. Polysaccharide-alone vaccines (purified Hib polysaccharide) were certified for use in the US in 1985 but suffered from an inability to elicit immunological memory, poor persistence of immunity, and poor immunogenicity in children under 2 years of age. Covalently coupling the polysaccharide to a protein carrier transformed the vaccine into T-dependent antigens and as such elicited strong immune responses, immunological memory, and immune responses in infants. The first approved HibCV in the US was manufactured using polyribosylribitol phosphate (PRP) conjugated to diphtheria toxoid (DT), though it was eventually replaced by more effective vaccines using meningococcal outer membrane protein (OMP), cross-reactive material 197 (CRM_197_), or tetanus toxoid (TT) carriers. HibCVs are usually used in combination with other pediatric vaccines including tetanus, diphtheria, hepatitis B, and pertussis (Table 1). Importantly, many developing country vaccine manufacturers (DCVMs) have licensed and secured WHO prequalification for low-cost Hib-containing vaccines, resulting in wide-spread introduction globally. The development, licensure, and introduction of HibCVs paved the way for other conjugate vaccines.

The prototypic pneumococcal vaccine was also polysaccharide-based, covering 23 serotypes, and was licensed in 1983 primarily for use in high-risk adults [11]. The first PCV (Prevnar^®^, PCV7) was licensed in the US in 2000 and was designed to protect against the seven most prevalent invasive disease serotypes in the US and Europe. PCV7 did not, however, protect against the serotypes responsible for considerable disease in LMICs, such as serotypes 1 and 5. Additionally, the introduction of PCV7 led to the emergence of non-vaccine serotypes, a phenomenon referred to as serotype replacement. This experience prompted the development of 10- and 13-valent PCVs that offer broader coverage (Table 1). Next generation PCVs that extend coverage up to 24 serotypes are currently in mid- to late-stage development.

The first licensed meningococcal vaccines were also polysaccharide based. More recently, NmCVs containing various combinations of serotypes A, C, W, and Y have been licensed and introduced (Table 1). There are two licensed vaccines for serotype B, both protein-based, though neither is WHO prequalified. Vaccines are in development that combine either serotypes A, C, W, X, and Y or serotypes A, B, C, W, and Y [12,13].

There are currently no licensed GBS vaccines, but several candidates are in early- to mid-stage clinical assessment, including a hexavalent version formulated with serotypes Ia, Ib, II, III, IV, and V undergoing Phase 1/2 clinical study [14]. A protein based GBS vaccine has advanced into multiple clinical studies and has demonstrated encouraging safety and immunogenicity data [15,16].

Conjugate vaccines are not without limitations, though; limited serotype coverage and serotype replacement have resulted in the need to make higher valency vaccines for pneumococcus and meningococcus. This, in turn, contributes to manufacturing complexity and difficulty in ensuring affordability for LMICs. Protein-based vaccines are a possible alternative for meningococcus and GBS, but the vaccine against serogroup B is the only protein-based vaccine (OMP/outer membrane vesicle [OMV]) currently licensed [17,18,19,20,21,22]. This approach is not needed for Hib as the current conjugate vaccines are effective, and it has been difficult to develop a protein-based vaccine for pneumococcus.

## 3. Early-Stage Meningitis Vaccine Development

The development of monovalent meningitis vaccines paved the way for newer licensed multivalent vaccines that have broader coverage, including the multivalent GBS vaccines currently under development. Multivalent conjugate vaccines (PCVs, NmCVs, and GBS conjugate vaccines) must be fit for purpose and are highly complex products from a manufacturing perspective—and, as such, are challenging from development and cost-effectiveness perspectives.

### 3.1. Considerations

The Target Product Profile or Preferred Product Characteristics are critical to guide early strategic decisions for all stages of meningitis vaccine development, from drug substance formulation to presentation, preclinical through clinical studies, product licensure, and introduction (Table 2). Additionally, WHO publishes technical report series (TRS) documents that provide guidance for assuring the quality, safety, and efficacy of vaccines—including for HibCVs, PCVs, and serogroup A and C NmCVs. The TRS includes recommendations on vaccine manufacturing, nonclinical evaluation, clinical evaluation, and for national regulatory authorities.

### 3.2. Manufacturing

Chemistry, Manufacturing and Controls (CMC) are well described for conjugate vaccines. Many vaccine attributes must be considered during development, including selection of the serotypes and carrier protein conjugation technology, formulation, presentation (Table 3), LMIC needs, and the cost of goods sold (COGS) [28]. The considerations to plan for include saccharide antigen, carrier protein, preservation of immunogenic epitopes, conjugation chemistry, stability of both drug substance and drug product, formulation, consistency of quality, analytics, preclinical models, and commercially viable manufacturing process. Due to the safety considerations for vaccines used in healthy humans, carrier protein choice is currently limited to CRM_197_, TT, OMP, DT, and *H. influenzae* protein D, each of which has nuances with antibody avidity and quantity of antibodies elicited.

Other considerations include the optimal methodologies for the fermentation and purification of polysaccharides and whether they should be native or size-reduced for conjugation. Several technologies have been used to conjugate the polysaccharides to the proteins in currently licensed meningitis vaccines, the most common of which involve reductive amination or cyanylation chemistry [28]. Newer technologies under development are designed to increase conjugation efficiency, simplify the manufacturing processes, and better preserve immunological epitopes on both the saccharide and protein components [32,33,34]. The use of an adjuvant is an important consideration and is often driven by either licensed vaccines or clinical assessment, as preclinical models are not good indicators of adjuvant benefits on immunogenicity.

### 3.3. Nonclinical Assessment

Evaluating meningitis conjugate vaccines in animal models provides an initial assessment prior to clinical evaluation. For licensed vaccines such as Hib, NmCVs, and PCVs, however, demonstrating protection against disease in a preclinical model is not required and assessment focuses on immunogenicity. Preclinical animal models usually differentiate between the antibody responses of the formulations being tested [28]. Cost, availability, study duration, cross-reactivity, and applicability to humans contribute to animal model selection, though ultimately the choice relies on published work on similar vaccines and compares the responses of the candidate vaccine to a licensed vaccine for the serotypes they have in common. In vivo experiments may not predict the human response but are the best way to distinguish between vaccine formulations. In vitro assays to measure antibody responses in the animal models are, ideally, identical to the assays used in human antibody evaluation. Non-human primates are sometimes used to evaluate immunogenicity for advanced candidates; however, they may not predict immune responses in humans [35,36]. Preclinical animal immunogenicity assessments and toxicology study data (to indicate safety if the product does not elicit a toxic response) are required by regulatory authorities prior to the first in-human clinical study.

If the vaccine is intended for maternal immunization, as is the case for GBS vaccines currently in development, or may be used in a campaign setting that includes pregnant women, as is the case for meningococcal vaccines, a developmental and reproductive toxicology study is required to understand the impact of the vaccine or vaccine candidate on fertility and developmental toxicity. Pre- and post-natal development studies are also necessary to understand the full spectrum of potential reproductive impacts.

### 3.4. Importance of Functional Assays

Preclinical and clinical measurements of immune responses to Hib, pneumococcus, meningococcus, and GBS conjugate vaccines have focused on binding and/or functional assays. Binding assays (typically enzyme linked immunosorbent assays) are simple, can be multiplexed, and are highly quantitative in nature. Additionally, it is critical to measure functional antibody responses, whether serum bactericidal activity (SBA) titers for NmCVs, or opsonophagocytic killing assay (OPK/OPA) titers for PCVs and GBS conjugate vaccines. Both SBA and OPK assays demonstrate the ability of vaccine-elicited antibodies to kill live bacteria and are considered to correlate better with clinical efficacy than IgG binding assays.

The use of standardized assays and reagents for both pre-clinical and clinical trial assessment is essential for comparing data between trials, establishing a correlate of protection, and understanding results in the absence of a comparator vaccine. Standardized assays and reagents exist for HibCVs, PCVs, and NmCVs and are in development for GBS conjugate vaccines [37,38,39].

### 3.5. Phase 1 Clinical Trials

Phase 1 trials obtain initial safety, reactogenicity, and immunogenicity data in healthy adults. When licensed vaccines exist, such as for HibCVs, PCVs, and NmCVs, the candidate vaccine is measured against a licensed one. Phase 1 studies may provide initial assessment of different dose levels and formulations both with and without adjuvant, though for HibCVs, PCVs and NmCVs these parameters are becoming well defined with multiple licensed products (Table 1). Notably, for conjugate vaccines, aluminum adjuvants are sometimes incorporated for vaccine stabilization rather than to enhance immune responses. Phase 1 trials are usually small (<100 subjects) so the dose range and adjuvant must be definitively assessed in a Phase 2 trial.

### 3.6. Phase 2 Clinical Trials

Phase 2 trials assess the dose selection, adjuvant need, safety, and antibody response to a licensed vaccine (when available) in a larger number of subjects in the target age group. This ensures sufficient statistical power to determine whether the vaccine is promising enough to advance to the next phase of clinical study. For GBS vaccines in development, immunogenicity will be assessed in pregnant women, in cord blood, and in the newborns to determine whether there is adequate transplacental transfer of antibodies and how well they persist.

## 4. Late-Stage Clinical Development

HibCVs, PCVs and NmCVs have followed distinct scientific and regulatory pathways in the late stages of their clinical development. However, their licensure strategies have certain aspects in common, based on similarities shared across the three targets, including the type of pathogen, the vaccine platform, and the clinical outcomes targeted. For instance, experience with conjugate vaccine technology allows developers to make initial assumptions regarding dose range and schedules for early clinical development and likely methods for immunological assessment. Similarly, all of these pathogens exhibit a wide spectrum of clinical disease, ranging from asymptomatic carriage to invasive disease, including sepsis and meningitis. Protection against these more severe conditions formed the basis for initial licensure of the early vaccine candidates—but the rare occurrence of these conditions in the population has had similar implications for subsequent vaccine development.

This last consideration has been one of the more consequential factors in shaping late-stage development of recent Hib, meningococcal, and pneumococcal vaccines. Licensure of the earliest conjugate vaccines was based on clinical efficacy trials against invasive bacterial disease outcomes, including meningitis, whose relatively low incidence required tens of thousands of participants. For instance, the efficacy of HibCVs was initially established through several randomized placebo-controlled clinical trials conducted in the late 1980s and early 1990s with invasive disease as the primary endpoint [40,41]. Conducted in both high-resource (California, UK, Finland) and lower-resource (Chile, The Gambia, US Alaskan Natives and Navajo) settings, these trials established the clinical efficacy of PRP conjugate vaccines based on four different protein carriers [41]. Having established the presence of safe and efficacious vaccines to protect against invasive Hib disease, it was considered unethical to conduct subsequent placebo-controlled efficacy trials that would leave a subset of participating infants unprotected. However, conducting a comparative efficacy trial between a new and an established vaccine would have been prohibitively large, given the low incidence of vaccine failures likely to occur in either arm. Therefore, later trials of HibCVs, either as new products, newer formulations (such as in combination vaccines), or in alternate schedules have relied on immunologic outcomes (anti-PRP serum IgG levels) for licensure.

In the case of meningococcal vaccines, the low incidence and sporadic epidemiology of disease in industrialized countries pushed this concept even further. The clinical efficacy of meningococcal vaccination was initially established with polysaccharide A and A/C vaccines more than 40 years ago. Effectiveness was demonstrated in closed populations of high-risk adults, demonstrating the vaccines’ utility in controlling outbreaks [42,43,44]. Later, when the UK became the first country to introduce NmCV (against serogroup C) in 1999, licensure was not granted on the basis of clinical efficacy, but rather on the demonstration of adequate immunogenicity [45]. The licensure of all subsequent NmCVs has been granted based on immunogenicity relative to an accepted surrogate of protection, with later demonstration of protection against clinical disease achieved following broader use [45]. Notably, this approach was used for vaccines containing additional meningococcal serogroups, including W and Y, despite having no studies linking specific antibody levels to clinical protection. Licensure was nevertheless granted based on the assumption that these conjugate vaccines would behave similarly, given the infeasibility of conducting efficacy trials for these serogroups. In contrast, serogroup B meningococcal vaccines were relatively delayed, as similarities between group B capsular polysaccharides and host epitopes prevented use of the polysaccharide conjugate platform. Instead, vaccines based on protein subunits were developed. Nevertheless, licensure was still granted based on the induction of serum bactericidal antibody, an immunological outcome, with a post-marketing commitment to demonstrate clinical benefit [46].

MenAfriVac^®^, a monovalent group A meningococcal conjugate vaccine (NmCV-A) developed through MVP (a partnership between WHO, PATH, and SIIPL), has been deployed through two strategies, first a series of national mass vaccination campaigns throughout the African meningitis belt covering a broad age group (1 to 29 years of age), followed by incorporation of the vaccine into the routine infant immunization (EPI) schedules of the affected countries. To accomplish this, the vaccine’s licensure strategy involved two stages. Initial licensure and WHO prequalification was based on a series of clinical trials in individuals 1 to 34 years of age demonstrating the safety and immunologic superiority of a full dose (10 µg PsA-TT) to a group A-containing polysaccharide vaccine [47,48,49], thus allowing the start of mass campaigns. Subsequently, an indication for a 5 µg single-dose regimen in children 3 to 24 months of age was achieved based on demonstration of immunologic non-inferiority to the 10 µg dose in two trials in infants [50].

More recently, the licensure strategy for a new pentavalent NmCV containing serogroups A, C, W, X and Y has followed a parallel path relying on demonstration of immunologic non-inferiority to established quadrivalent conjugate vaccines. Two ongoing Phase 3 trials, one in 2- to 29-year-old individuals in Mali and The Gambia [51,52,53,54], and another in adult and elderly individuals in India, both using Menactra as the comparator, are intended to gain licensure for use in mass campaigns and travelers. Another Phase 3 trial is planned for younger infants and toddlers in Mali to allow use in routine infant immunization. This trial will use Nimenrix as the comparator because, unlike Menactra, Nimenrix is licensed for use as a single dose down to 6 months of age.

Finally, for PCVs, the clinical efficacy of initial 7- and 9-valent vaccines against invasive pneumococcal disease (IPD) was established in four large-scale trials conducted in the late 1990s and early 2000s in both high- and low-income settings [52,53,54,55]. The observed efficacy in these studies ranged between 76.8 and 97.4 percent for IPD caused by serotypes contained in the vaccine, with higher efficacy seen in more industrialized settings. A later 10-valent vaccine was initially licensed using immune correlates of protection, with effectiveness subsequently established through two randomized double-blind controlled trials in the late 2000s in Finland (in a cluster-randomized design) and Latin America [56,57]. Vaccine development expanding the initial 7-valent vaccine to a 13-valent formulation and comparisons for different immunization schedules for the PCV13 and PCV10 vaccines subsequently relied on immunologic endpoints [58], as did the development and licensure of a newer 10-valent PCV in India [59].

In the evaluations of efficacy noted above, the clinical endpoints were chosen by balancing a need for the specificity and clinical relevance of laboratory-confirmed severe disease with the practicality of measuring relatively uncommon outcomes in a population. By necessity, meningococcal vaccine trials were limited to evaluation of protection against meningitis in the case of polysaccharide vaccines, and immunologic outcomes for conjugate vaccines. For Hib and pneumococcal vaccines, initial clinical trials assessed efficacy against all invasive disease, including bacteremia, bacteremic pneumonia, and meningitis, typically in such low numbers that these presentations were not differentiated in their reporting. The effectiveness of these vaccines in the prevention of meningitis specifically has been demonstrated in multiple later studies following implementation in various countries.

An important consideration for the overall clinical development plan as specified in WHO TRSs is the incorporation of antibody persistence studies to inform vaccine implementation strategies and schedules that may potentially require booster doses. For example, in the case of NmCV-A, antibody persistence analysis was used to estimate that protective immune responses would persist for at least 10 years following immunization [60]. As mentioned earlier, a critical feature of conjugate vaccines is their ability to invoke herd protection. The ability to prevent acquisition of carriage, an indicator for herd immunity, can be assessed in Phase 3 trials or in post-licensure studies.

### Immunological Correlates of Protection

Despite the similarities among these vaccines, there are also aspects that were unique or assumed special prominence for each pathogen. Ideally, the reliance on immunologic endpoints for regulatory or policy decision-making should be based on a true immune correlate of protection. However, such a correlate is not always available. In the case of Hib vaccines, two immunologic correlates were established. Based on initial experimental data, an anti-PRP IgG level of 0.15 µg/mL indicated ongoing protection from invasive Hib disease, while field studies indicated that a peak post-vaccination response level of 1.0 µg/mL was needed for long-term protection (Table 4). As a result, both thresholds were ultimately considered for regulatory approval and post-licensure evaluation of new vaccines and schedules [41]. The presence of immune correlates proved to be particularly useful for assessing the adequacy of different infant schedules, especially those that were accelerated (2, 3, and 4 months) or early (6 weeks) [61]. Immune correlates were also instrumental in evaluating potential immunological interference between Hib and other childhood vaccines. For instance, a resurgence of Hib cases in the UK in the early 2000s was attributed to interference between Hib vaccine and the recently adopted acellular pertussis vaccines. Evaluation of antibody levels in cohorts receiving both vaccines revealed lower anti-PRP IgG levels in later toddler years compared to prior cohorts, prompting the addition of a Hib booster dose at school entry [41]. Benchmarking antibody levels to short- and long-term thresholds became prominent again in subsequent years, as more complex combination infant vaccines were developed. Immunologic evaluation of these formulations revealed not only interactions between Hib, other antigens, and their carrier proteins, but also incompatibilities among adjuvants [62]; nevertheless, multiple Hib-containing pentavalent and hexavalent vaccines have ultimately come to market.

For meningococcal vaccines, maintaining adequate levels of circulating serum antibody is considered most important, as the onset of severe clinical disease upon exposure is too rapid to allow time for generation of an immune memory recall response [45,65]. Therefore, assuring serum antibody persistence has been an important feature of meningococcal vaccine evaluation. The immunological evaluation of NmCVs has focused on functional immune responses, namely SBAs. In comparison with HibCVs or PCVs, NmCVs require only one or two doses for durable protection, which may be partly due to the older ages at which they are generally given [42].

In the case of pneumococcal vaccines, a meta-analysis of humoral responses using pooled results from three of the original efficacy trials was conducted, allowing the scientific community to establish a non-inferiority threshold of 0.35 µg/mL capsular polysaccharide antibody against each serotype for the evaluation of newer PCVs. While this threshold is not serotype-specific, and true correlates of protection for specific serotypes may ultimately vary [66], this benchmark has allowed the development of later PCV formulations with higher valency based on immunologic outcomes [67].

Among the major causes of bacterial meningitis, GBS has remained a challenge for vaccine developers. Notably, the early age at which this pathogen acts indicates the best approach to vaccination would be administration during pregnancy to transfer protection to the infant through maternal antibody. While regulatory guidance has been proposed for this novel indication, no “maternal” vaccine has yet been licensed for this purpose, and several uncertainties remain, particularly regarding late-stage development [68].

Several GBS vaccine candidates are currently in Phase 2 development, and progression to licensure will follow one of two main pathways: efficacy trials demonstrating protection against specific clinical outcomes, or immunogenicity trials that target immunologic correlates of protection. Each developmental program has its own strengths and challenges.

Demonstration of clinical efficacy through randomized controlled trials would be the most direct route to licensure. As with the other pathogens discussed in this review, GBS is associated with a wide spectrum of disease, with laboratory-confirmed invasive disease (early- and late-onset meningitis being particularly prominent) the most likely clinical endpoint, given its specificity and relevance to clinical care and public health [69]. Similarly, this outcome is relatively uncommon, particularly if focused on neonatal disease alone, and thus would require relatively large clinical trials to establish efficacy. For this reason, composite endpoints that incorporate additional important laboratory-confirmed fetal and obstetric outcomes, such as stillbirth and maternal sepsis, have been proposed to reduce study size [69]. Neonatal invasive GBS disease occurs at a rate of 1 to 3 per 1000 live births in many geographies, and in those areas, best practices associated with prenatal and perinatal care and intrapartum antibiotic prophylaxis can reduce this rate to 0.5–1.0 per 1000 live births. Given these incidence rates, an efficacy trial could require between 30,000 and 1.8 million mother-infant pairs [39]. While some infant vaccine trials have included up to 70,000 participants, evaluating maternal immunization would also be more resource-intensive on a per-subject basis by comparison. Other clinical endpoints could be considered, including maternal urinary tract infection and colonization, but are unlikely to be included, as they do not directly correlate with invasive disease and otherwise do not pose a significant clinical or public health burden.

Given the impracticality of conducting clinical trials of this size, developers must consider pathways that utilize an immunologic endpoint. However, without prior vaccine efficacy trials, a correlate of protection must be established through sero-epidemiological studies that examine naturally occurring disease. Since the 1970s, serotype-specific maternal capsular antibodies were known to correlate with a reduced risk of invasive GBS disease. However, differences in methodology prevented the establishment of protective thresholds. More recently, larger-scale studies have been initiated in South Africa and the UK using a standardized approach to more definitively establish these associations. These efforts, along with data from animal models, will hopefully produce suitable criteria for pivotal Phase 3 vaccine trials based on immunologic endpoints [39].

Several aspects of the immune response to vaccination are particularly relevant to the maternal immunization model. Since fetal and infant protection is primarily generated through passive transfer of IgG antibody through the placenta during gestation, achieving a high peak maternal serum IgG antibody response to maximize infant levels by the time of birth is a key objective. Therefore, longevity of the immune response, generation of durable immune memory, and even protection of the mother, are secondary—although important—goals. In addition, since this model involves adult vaccine recipients who likely have been previously exposed to GBS, a single vaccine dose to boost pre-existing memory responses is likely to be sufficient. Finally, either before or after licensure, vaccine manufacturers will need to demonstrate a lack of immune interference between their GBS vaccine and other vaccines currently given to pregnant women, including tetanus, pertussis, and influenza, or under development, such as respiratory syncytial virus. Moreover, compatibility studies among these vaccines could allow their incorporation into a combination maternal vaccine, which could greatly improve affordability and access.

## 5. Accelerating Vaccine Introduction to Prevent Meningitis

Introducing HibCVs, NmCVs, and PCVs and optimizing their coverage in affected populations has been critical for reducing meningitis morbidity and mortality in the last 20 years. However, the availability of effective and safe vaccines alone is insufficient to increase LMIC uptake. Despite the success of these vaccines in high-income countries, overcoming barriers to introduction and sustaining vaccine delivery in LMICs—where the greatest meningitis burden persists—remains a major challenge to global meningitis control [70].

### 5.1. HibCV: Developing New Approaches to Increase Meningitis Vaccine Uptake

In 2000, 13 years after HibCV was licensed, Hib still caused 8 million meningitis cases and about 400,000 deaths in u5 children in LMICs [71,72]. No Asian countries and only one sub-Saharan African country had introduced HibCV. By 2008, 70 percent of WHO members had introduced HibCV; Hib deaths in u5 children were cut in half [73]. Despite this remarkable impact, HibCV uptake remained low in LMICs. New LMIC introduction approaches were needed.

In the late 1990s, public-private partnerships started to develop new policies, strategies, and priorities for vaccine introduction and to financially support HibCV procurement—dramatically increasing uptake in LMICs [74]. In 2006, combining HibCV into WHO-prequalified quadri-, penta-, and hexavalent vaccines accelerated uptake and contributed to sustain HibCV use in Gavi-eligible countries [75]. These strategies and approaches would be replicated to increase uptake of NmCV-A and PCVs (Table 5). Incorporating similar approaches will lead to successful introduction of GBS vaccines and boost uptake of multivalent NmCVs and higher valency PCVs. In addition, as countries become ineligible for Gavi support, three approaches (vaccine procurement groups; lower-price, high-quality, WHO-prequalified vaccines from DCVMs; and combination vaccines) will allow middle-income countries (MICs) to continue to introduce new meningitis vaccines.

### 5.2. Defining Meningitis Burden to Justify Vaccine Introduction

Poor understanding of the meningitis burden is a major hurdle that requires considerable time, effort, and resources to overcome. Laboratory-based meningitis surveillance to identify at-risk populations, detect outbreaks, and define the potential impact of meningitis control shows the public health value of these vaccines. Meningitis surveillance is challenging and requires significant technical capacity to culture blood/cerebrospinal fluid and identify serogroups/serotypes of meningitis pathogens. However, without evidence that a specific pathogen is a public health problem, countries will be slow to commit to vaccine introduction.

The highest meningococcal disease burden is in the 26 countries of the African meningitis belt. From 1970 through 2010, recurring explosive serogroup A meningococcal (Nm-A) meningitis epidemics in sub-Saharan Africa increased in frequency and magnitude [77]. In 1992, WHO country offices, UNICEF, and non-governmental organizations (NGOs) began submitting outbreak data to WHO to justify release of stockpiled meningococcal vaccines. Because these periodic Nm-A epidemics largely defined the meningitis burden, these surveillance data-containing requests yielded data to support NmCV-A introduction. In 2014, MenAfriNet, a case-based meningitis surveillance system, began monitoring meningitis outbreaks, which will be important in future decisions to introduce a multivalent NmCV.

Hib meningitis results from endemic transmission. Because u5 children accounted for 90 percent of Hib meningitis cases and parents often seek hospital care for ill children, hospital-based surveillance of 0- to 59-month-old children was used to define disease burden [78]. Because only one serotype caused disease, the laboratory demands were much less than those for meningococcus and pneumococcus. This surveillance resulted in high quality burden data in Africa, where Hib was well recognized as a meningitis pathogen. However, most Asian countries did not show sufficient burden to justify HibCV introduction; that changed when a landmark vaccine probe study in Indonesia showed that Hib accounted for a large portion of meningitis and pneumonia not found in routine surveillance [79,80]. Subsequent vaccine probe studies showed significant reduction of meningitis was possible through vaccination and greatly accelerated HibCV uptake [81].

In LMICs that successfully introduce HibCV and NmCV-A, pneumococcus becomes the most common cause of meningitis in all age groups—yet, defining pneumococcal meningitis burden can be difficult [82]. Because of the disease’s endemic transmission, broad age distribution, and multiple serotypes, defining the best surveillance is a challenge. As a result, pneumococcal meningitis burden data are often underestimated and insufficient alone to justify PCV introduction. It is better justified by the much higher burden of community-acquired pneumococcal pneumonia and PCVs’ cost-effectiveness in preventing pneumonia. Compared to the pneumonia burden, except for periodic serotype 1 pneumococcus meningitis epidemics in Africa, pneumococcal meningitis surveillance has played a small role in accelerating PCV uptake.

### 5.3. Highly Directive Policies from Global Public Health Authorities

Global public health authorities have highly influential voices that can be used to advance vaccine introduction. Because of the challenges in diagnosing Hib meningitis, its high treatment costs, high mortality, and the severe neurologic impacts in survivors, HibCV was clearly cost-effective in most LMICs [83]. Yet, decisions to introduce HibCV lagged for many reasons, including inadequate in-country technical capacity to assess the value and potential impact of vaccines [84]. In 2006, WHO overcame this barrier when it universally recommended the implementation of Hib vaccination in all infant immunization programs worldwide without accumulating more surveillance data [76]. Such a statement was possible because the global risk of Hib meningitis was roughly the same for all children, the potential impact of vaccination was similar globally, and HibCV had an excellent safety and efficacy profile [81]. This statement was critical in the LMIC decisions to introduce HibCV [85].

### 5.4. Structuring Vaccination Strategies for Success

Successful vaccine introduction strategies can have a high impact in a short time and can motivate decision-makers in other countries to introduce new vaccines. Successful introduction strategies begin by clearly defining target populations. Several factors come into play when defining this target, such as peak-incidence age, opportunities to vaccinate, persistence of immunity, and need for a booster vaccine. HibCV and NmCV-A introduction showed that well-targeted strategies can quickly achieve near elimination of disease and that successful introduction in early-adopting countries led to decisions to introduce in other countries.

The vaccine introduction strategy for HibCV was relatively straight-forward. Because WHO recommended vaccination for all children, there was no need to develop surveillance systems to identify at-risk countries, districts, or populations or to develop subnational introduction plans. Because peak-incidence age was in the first two years of life, vaccine had to be delivered to infants, and national immunization programs had well-developed opportunities to vaccinate 6-, 10-, and 14-week-old infants.

In contrast, NmCV-A does not universally benefit all children because Nm-A meningitis is not equally distributed globally [86]. Although epidemics were reported globally until the 1940s, Nm-A meningitis outbreaks had become restricted to African meningitis belt countries. Moreover, Nm-A meningitis was not equally distributed within countries. Consequently, highly granular disease surveillance data was needed to allow subnational NmCV-A introduction. Whereas the goal of HibCV was to prevent endemic disease, the goal of NmCV-A was to prevent periodic epidemics driven by meningococcal nasal carriage in children 10 to 14 years of age. The decision to conduct introduction campaigns in 1- to 29-year-olds was a strategy that stopped outbreaks and prevented meningitis in young adolescents. However, this strategy had to be balanced by the fact that routine vaccine delivery to school-aged children is not well-developed. Currently, NmCV-A vaccination occurs in children younger than 2 years of age. Whether bactericidal antibodies persist beyond 10–12 years at a level that will later suppress nasal carriage and prevent Nm-A epidemics is unknown.

### 5.5. Public-Private Initiatives to Provide Vaccine and Improve Vaccination Practices

Public-private partnerships have been critical to HibCV, NmCV-A, and PCV introduction by providing support for vaccine delivery, procurement, and technical assistance to low-income countries (LICs). These partnerships will remain critical for new vaccine introduction going forward.

In 1998, the William H. Gates Foundation donated $100 million to establish the Children’s Vaccine Initiative (CVI) to improve vaccine delivery to LICs [87]. Prior, many LICs used funds intended to support delivery to buy HibCV. To reverse this, CVI proposed funding to make vaccines more available and to improve the quality of vaccine delivery, rather than to procure vaccines. Through partnerships with WHO, UNICEF, PATH, and other international NGOs, CVI funded guideline development, vaccination worker training, model immunization programs, cost-effectiveness studies, and advocacy and communication programs to increase HibCV acceptance.

In 2001, the Bill & Melinda Gates Foundation funded MVP, which successfully developed, tested, licensed, WHO-prequalified, and introduced MenAfriVac^®^, an affordable NmCV-A. The keys to MVP’s success included developing strong public private partnerships [7,88,89]; engaging SIIPL, a DCVM, to develop a low-cost, high-quality NmCV-A; providing technical assistance to SIIPL to acquire WHO prequalification; conducting clinical trials in Africa alongside African researchers; and supporting operational costs of introduction. Since 2010, more than 340 million Africans have been vaccinated with NmCV-A and Nm-A meningitis has been eliminated from this region.

In 2002, Gavi and key partners, including Johns Hopkins University, established the Pneumococcal Vaccines Accelerated Development and Introduction Plan (PneumoADIP) to increase uptake of PCVs in Gavi-eligible countries [90]. The keys to PneumoADIP’s success included supporting PCV procurement and the operational cost of vaccine introduction, standardizing pneumococcal disease surveillance, developing advocacy and education activities to inform country decision-makers within national immunization programs regarding PCV and HibCV introduction, and providing technical assistance for vaccine introduction campaigns and the transition to routine immunization. As a result of PneumoADIP, between 2000 and 2018, 59 of 73 Gavi-eligible countries introduced PCV.

In 2005, Gavi’s Hib Initiative (GHI), a consortium of WHO, Johns Hopkins University, London School of Hygiene and Tropical Medicine, and the Centers for Disease Control and Prevention, was funded to help Gavi-eligible countries make evidence-based decisions regarding HibCV introduction [85]. Through these CVI and GHI activities, the number of LICs introducing HibCV increased from 13 in 2004 to 66 in 2008 [91]. Currently, all Gavi-eligible countries use HibCV-containing vaccines.

### 5.6. Developing Innovative Vaccine Financing Options

Defining the cost-effectiveness of HibCV, NmCV-A, and PCVs has been important for new vaccine decision-makers and has accelerated the uptake of these vaccines in LMICs. Studies have shown that HibCV is cost saving or highly cost-effective in essentially all settings. Cost-effectiveness has further increased due to the recent decline in HibCV prices, integration of HibCV into quadri-, penta- and hexavalent combination vaccines, and data showing the loss of productivity in meningitis survivors [92]. Similarly, compared with a reactive vaccination strategy, prevention strategies using NmCV-A were shown to be significantly cost saving in Burkina Faso [93]. Such analyses will be important for decision-makers considering whether the higher price of the next generation of meningococcal or pneumococcal vaccines or the price of new vaccines to prevent GBS meningitis are justified by their benefits [94].

Prior to 2000, vaccine cost was often the greatest barrier to meningitis vaccine introduction. Since then, LICs have greatly benefited from Gavi’s vaccine investment strategy and procurement of meningitis vaccines through The Vaccine Fund [95,96]. Unfortunately, many MICs that procure their own vaccines face financial challenges to introduction. In addition, LIC decision-makers are more widely considering the long-term costs of vaccination, not just the initial introduction costs.

To address this, in 2009, the Advance Market Commitment (AMC) for pneumococcal vaccines was launched. In the AMC, donors commit funds to guarantee the price of vaccines once they have been developed. In exchange, manufacturers make a legally binding commitment to provide the vaccines at a price affordable to LICs [97]. Although the AMC has been recognized as a valuable way to make effective and affordable pneumococcal vaccines available, it has also been criticized for not encouraging innovation, discouraging competition from new market entrants, and raising vaccine costs [98,99].

Another financing option is multi-country procurement groups, such as the Pan American Health Organization (PAHO) Revolving Fund. Since 1977, this fund has pooled the resources of 41 mostly middle-income Latin American countries to procure vaccines at a lower cost through consolidated ordering [100]. Currently, the fund is used to procure HibCV-containing vaccines and PCV, which has resulted in sustained use of these vaccines throughout Central and South America.

Finally, for countries that purchase their own vaccines, the availability of lower-cost, high-quality, WHO-prequalified vaccines produced by DCVMs has been an important alternative to vaccines produced by multi-national vaccine manufacturers.

### 5.7. Implications for New Meningitis Vaccines

The lessons learned from HibCV, NmCV-A, and PCV introduction will likely be applied to the introduction of new meningitis vaccines. For example, there has been development and successful use of several other meningococcal vaccines, including monovalent meningococcal vaccines against serogroups C and B and multivalent NmCVs against serogroups A, C, W, X and Y. WHO has stated the decision to use other meningococcal vaccines or to replace NmCV-A with a multivalent NmCV will depend on the locally prevalent meningococcal serogroup(s), identification of the best target group for vaccination, and opportunities to vaccinate within national immunization programs [42]. This underscores the importance of meningitis surveillance. Discussion is ongoing regarding the use of new multivalent NmCVs being developed by DCVMs.

New vaccines are being developed against GBS to prevent meningitis in neonates and young infants [27]. Some of the approaches described above will likely be used to increase uptake (e.g., combination vaccines, support for vaccine procurement) [27]. However, because the goal of a GBS vaccine is to prevent invasive disease in neonates and infants, the target group for vaccination is pregnant women. Given the challenges of accessing obstetric care in LICs and the lower emphasis on vaccination in antenatal care clinics compared to EPI clinics, new approaches will be needed with special attention to advocacy and communication and antenatal healthcare worker training to introduce a GBS vaccine.

## 6. Conclusions and Future Directions

The development and global introduction of low-cost vaccines to prevent Hib and pneumococcus has had a significant impact on meningitis and other disease manifestations caused by these pathogens. DCVMs have become the major suppliers of affordable Hib combination vaccines and the recent licensure and WHO prequalification of a 10-valent PCV by SIIPL, in partnership with PATH, is poised to increase availability of low-cost PCVs for LMICs, notably in those countries that have not introduced PCVs into their routine immunization programs. Like with Hib vaccines, it is anticipated other DCVMs will license PCVs and increase the global supply of affordable vaccines. Despite the considerable success in reducing the burden of pneumococcal disease globally, serotype replacement and emergence has resulted in significant residual disease burden. Higher valency (15–24 serotypes) PCVs are in development, though there are considerable manufacturing and licensing challenges for such vaccines and LMIC affordability is uncertain.

Meningococcal vaccines present a dichotomy: Quadrivalent NmCV-ACWY and meningococcal serogroup B protein vaccines manufactured by multinational vaccine manufacturers are cost prohibitive for widespread use in LMICs, while a low-cost NmCV-A that has had incredible impact in the African meningitis belt has limited utility in other parts of the world. The development and licensure of low-cost NmCV-ACWY(X) and meningococcal B vaccines has the potential for broad appeal and to greatly reduce the burden of meningococcal meningitis globally.

In addition to reducing the per dose cost of meningitis vaccines, strategies to increase cost-effectiveness by minimizing the number of doses administered are in development. For example, WHO currently recommends a single dose of NmCV-A at 9 to 18 months of age for routine immunization and studies to assess whether a 2-dose schedule (1 + 1) instead of 3-dose schedule for PCVs may be sufficient to maintain adequate herd immunity are underway [101,102].

What about other meningitis pathogens that are potentially vaccine preventable? *Haemophilius influenzae* type A (Hia) causes meningitis in certain regions and populations globally, including indigenous populations in North America and Australia. Development of a Hia vaccine should be technically feasible but a limited market would likely require donor support to incentivize a manufacturer. *Klebsiella pneumoniae* is becoming increasingly recognized as an important cause of sepsis and meningitis in neonates in LMICs and as such could be targeted for maternal vaccine together with GBS. The relatively high number of *K. pneumoniae* capsular serotypes makes this a challenging approach, although targeting a more limited number of O antigens or protein antigens is also being considered [103].

Defeating meningitis is an ambitious undertaking that will require significant time, effort, and resources—particularly when it comes to developing new or improved meningitis vaccines. There are hurdles along the vaccine development and delivery spectrum but well-established vaccines like HibCV, PCV, and NmCV offer lessons for what does and does not work, how to successfully advance products toward market, and how to ensure they reach the populations in need—and where gaps remain that need to be filled. Despite their challenges, vaccines are a public health best-buy and have been critical to the progress we have made against meningitis thus far. Vaccines have saved millions of lives around the world and new entrants are poised to take that success further to make the vision of defeating meningitis by 2030 a reality.

## Figures and Tables

**Table 1:** World Health Organization (WHO) prequalified Hib, pneumococcal, and meningococcal conjugate vaccines [23].

Disease	Vaccine	Manufacturer
Hib	Monovalent (Hib)	Centro de Ingenieria Genetica y Biotecnologia, Sanofi Pasteur, Serum Institute of India, Pvt. Ltd. (SIIPL)
	Quadrivalent (DTP, Hib)	SIIPL
	Pentavalent (DTP, Hep B, Hib)	SIIPL, BioFarma, Biological E, LG Chem, Panacea, Sanofi India (Shantha)
	Pentavalent (DTP, polio, Hib)	Sanofi Pasteur
	Hexavalent (DTP, Hep B, polio, Hib)	Sanofi Pasteur
Pneumococcal	13-valent	Pfizer
	10-valent	GlaxoSmithKline (GSK), SIIPL
Meningococcal	Men A monovalent	SIIPL
	Men A, C, W, Y quadrivalent	GSK, Pfizer, Sanofi Pasteur

**Table 2:** Components of a meningitis vaccine Target Product Profile [24,25,26,27].

Attribute	General Considerations
Indication	Prevention of invasive disease (including meningitis) by HibCV, PCV, NmCV, and GBS. PCVs also indicated for pneumonia and otitis media.
Target population/age groups	HibCV: Infants and children u5. PCV: Generally infants and children u5. NmCV: Infants, children, and adolescents. GBS: Pregnant women to protect infants ≤ 3 months of age.
Serotypes	Invasive disease serotypes based on epidemiology of countries/populations targeted.
Immunogenicity	Assays used by licensed vaccines to measure IgG and functional responses (SBA ^a^ and OPK ^b^). Clinical trials should include a persistence timepoint and ensure the data package is rigorous per TRS recommendations.
Safety, reactogenicity, and contraindications	Similar to other licensed conjugate vaccines; incorporating TRS recommendations.
Schedule	As recommended by WHO and countries’ national immunization program schedules.
Interference and co-administration with other vaccines	Phase 2/3 studies should assess safety and immune responses to vaccines co-administered in target population per WHO and country EPI requirements.
Route of administration	Typically intramuscular. Other routes (e.g., intradermal and mucosal) can be considered.
Product presentation	Useable in target countries (prefilled syringes/vials/liquid/lyophilized). Multidose could reduce cost per WHO recommendations.
Product formulation	Attributes include stability, consistency in quality, manufacturability. Preservative may be necessary for multidose formulations targeted toward LMICs. Aluminum based adjuvants used for some conjugate vaccines.
Storage and cold chain requirements	Address storage and cold chain options in target countries; shelf life, temperature, ability to stockpile.
Packaging and labeling	Translate to local language.
Product registration and WHO prequalification	Advanced planning essential to ensure data package is appropriate for the regulatory agency and WHO (if prequalification is the goal).
Post marketing surveillance	Monitor safety, protection of target population, potential serotype replacement, and herd immunity.
Value proposition	Marketing attributes that contribute to the product’s business case (commercial interest, advantage over licensed products, cost to produce, etc.).

^a^ serum bactericidal activity, ^b^ opsonophagocytic killing.

**Table 3:** CMC general considerations [28,29,30,31].

Manufacturing Recommendations	Comments
Polysaccharide (s): Strains Seed lots Culture medium Purification Release testing	Source and identity. High yield strains. Master and working cell banks. Animal product free medium highly desirable.
Carrier protein (s): Source Purity Release testing	Commonly tetanus toxoid, diphtheria toxoid, CRM_197_, and OMP/OMV. CRM_197_ can either be native (expressed in *Corynebacterium diphtheriae*) or recombinant.
Monovalent bulk (s): Conjugation chemistry Release testing	Efficient conjugation contributes to lower COGS.
Final bulk: Adjuvant formulation (as needed)	
Filling and containers	Multidose liquid presentations require a preservative.
Control tests on final product	Stability indicating assays are key—typically free polysaccharide and size distribution.

**Table 4:** Immunological correlates of protection for Hib, meningococcal, pneumococcal, and group B streptococcus (GBS) vaccines [37,38,39,63,64].

Vaccine	Correlate of Protection	Notes
PCV	IgG concentration of ≥ 0.35 µg/mL.	Weighted data across serotypes from 3 efficacy studies. Individual serotypes vary (Goldblatt).
HibCV	IgG concentration of ≥ 0.15 and ≥1 µg/mL.	Immediate and long-term protection.
NmC	hSBA ^a^ of ≥ 4 or Rsba ^b^ ≥ 8.	
Other Nm serogroups	Correlates not defined but thresholds the same as NmC often used.	
GBS	Proposed to be between 1 and 10 µg/mL in pregnant women.	Assay standardization in progress.

^a^ Human complement serum bactericidal activity, ^b^ rabbit complement serum bactericidal activity.

**Table 5:** Introducing polysaccharide conjugate vaccines to prevent meningitis due to Hib, meningococcus, and pneumococcus in LMICs [1,29,58,76].

Vaccine	Meningitis Epidemiology	WHO Introduction Recommendation	Specific Vaccination Strategy			Activities to Accelerate Uptake and Sustained Use
			Countries	Within countries	EPI schedule	
HibCV	Peak incidence: <2 years of age. Endemic transmission.	All children, all countries. Initially, RI ^1^ for children <4 mo. of age.	All	National	Multiple, multi-dose RI schedules; first dose critical by 6 weeks to 2 mo. of age.	(1) Highly proscriptive WHO recommendation. (2) Vaccine procurement through The Vaccine Fund and Gavi. (3) Use of vaccine probe studies. (4) Develop HibCV containing penta- and hexavalent combination vaccines.
NmCV-A	Peak incidence: 9 to 14 years of age. Low-level endemicity with periodic outbreaks.	Meningitis Belt residents. Initially, SIA ^2^ for persons 1 to 29 years of age, then RI in children 9 to 18 mo. of age.	Epidemic prone, African countries.	Mix, national and subnational.	1-dose primary in children 9 to 18 mo. of age. Need for booster dose not yet determined. Strategy to use NmCV-5 is being developed.	(1) Enhanced surveillance linking emergency vaccine requests to define meningitis burden. (2) Develop low-price, high-quality, 1-dose vaccine through DCVM.
PCV	Peak incidence: ~85 percent of pneumococcal meningitis cases occur in children <2 years of age. Endemic transmission.	Initially, RI for <6 mo. of age.	All	National	Currently, for children <6 mo. of age: (1) 3-dose primary/no booster. (2) 2-dose primary/booster at 9 to 18 mo.	Unique financing options (e.g., Advance Market Commitment, PAHO Revolving fund).

^1^ RI–routine administration within EPI schedule; ^2^ SIA–supplementary immunization activity (mass campaign).
